# Non-invasive assessment of glioma microstructure using VERDICT MRI: correlation with histology

**DOI:** 10.1007/s00330-019-6011-8

**Published:** 2019-03-19

**Authors:** Fulvio Zaccagna, Frank Riemer, Andrew N. Priest, Mary A. McLean, Kieren Allinson, James T. Grist, Carmen Dragos, Tomasz Matys, Jonathan H. Gillard, Colin Watts, Stephen J. Price, Martin J. Graves, Ferdia A. Gallagher

**Affiliations:** 1 Department of Radiology, School of Clinical Medicine, University of Cambridge, Box 218, Cambridge Biomedical Campus, Cambridge, CB2 0QQ UK; 2 Department of Radiology, Cambridge University Hospitals NHS Foundation Trust, Cambridge, UK; 3 Cancer Research UK Cambridge Institute, University of Cambridge, Cambridge, UK; 4 Department of Pathology, Cambridge University Hospitals NHS Foundation Trust, Cambridge, UK; 5 Institute of Cancer and Genomic Sciences, Birmingham Brain Cancer Program, University of Birmingham, Birmingham, UK; 6 Neurosurgery Unit, Department of Clinical Neurosciences, University of Cambridge, Cambridge, UK

**Keywords:** Diffusion magnetic resonance imaging, Diagnostic imaging, Glioma, Brain neoplasms, Cancer

## Abstract

**Purpose:**

This prospective study evaluated the use of vascular, extracellular and restricted diffusion for cytometry in tumours (VERDICT) MRI to investigate the tissue microstructure in glioma. VERDICT-derived parameters were correlated with both histological features and tumour subtype and were also used to explore the peritumoural region.

**Methods:**

Fourteen consecutive treatment-naïve patients (43.5 years ± 15.1 years, six males, eight females) with suspected glioma underwent diffusion-weighted imaging including VERDICT modelling. Tumour cell radius and intracellular and combined extracellular/vascular volumes were estimated using a framework based on linearisation and convex optimisation. An experienced neuroradiologist outlined the peritumoural oedema, enhancing tumour and necrosis on T2-weighted imaging and contrast-enhanced T1-weighted imaging. The same regions of interest were applied to the co-registered VERDICT maps to calculate the microstructure parameters. Pathology sections were analysed with semi-automated software to measure cellularity and cell size.

**Results:**

VERDICT parameters were successfully calculated in all patients. The imaging-derived results showed a larger intracellular volume fraction in high-grade glioma compared to low-grade glioma (0.13 ± 0.07 vs. 0.08 ± 0.02, respectively; *p* = 0.05) and a trend towards a smaller extracellular/vascular volume fraction (0.88 ± 0.07 vs. 0.92 ± 0.04, respectively; *p* = 0.10). The conventional apparent diffusion coefficient was higher in low-grade gliomas compared to high-grade gliomas, but this difference was not statistically significant (1.22 ± 0.13 × 10^−3^ mm^2^/s vs. 0.98 ± 0.38 × 10^−3^ mm^2^/s, respectively; *p* = 0.18).

**Conclusion:**

This feasibility study demonstrated that VERDICT MRI can be used to explore the tissue microstructure of glioma using an abbreviated protocol. The VERDICT parameters of tissue structure correlated with those derived on histology. The method shows promise as a potential test for diagnostic stratification and treatment response monitoring in the future.

**Key Points:**

*• VERDICT MRI is an advanced diffusion technique which has been correlated with histopathological findings obtained at surgery from patients with glioma in this study.*

*• The intracellular volume fraction measured with VERDICT was larger in high-grade tumours compared to that in low-grade tumours.*

*• The results were complementary to measurements from conventional diffusion-weighted imaging, and the technique could be performed in a clinically feasible timescale.*

**Electronic supplementary material:**

The online version of this article (10.1007/s00330-019-6011-8) contains supplementary material, which is available to authorized users.

## Introduction

Gliomas are primary brain tumours characterised by diffuse infiltration and a poor prognosis [1, 2]. Genetic heterogeneity and phenotypic heterogeneity contribute to both poor therapy response and tumour recurrence [3, 4]. Magnetic resonance imaging (MRI) is the imaging modality of choice but is limited in assessing tumour subtypes and intratumoural heterogeneity [5–8]. In addition, conventional imaging is ineffective in evaluating the spread of tumour into the peritumoural region which may lead to sub-optimal resection and recurrence [9]. Consequently, new imaging biomarkers are required to improve the assessment of glioma.

Several studies have addressed the importance of diffusion-weighted imaging (DWI) and the apparent diffusion coefficient (ADC) in evaluating gliomas but have shown a partial overlap in the measured values between subtypes [10–12]. The ADC may have a role in differentiating high-grade glioma from metastases and in assessing peritumoural oedema, but it cannot accurately differentiate primary brain tumour subtypes [11–15]. The vascular, extracellular and restricted diffusion for cytometry in tumours (VERDICT) is a model that infers tissue microstructure from DWI measurements [16]. This model derives multiple compartments (intracellular, intravascular and extracellular–extravascular spaces), has been applied to xenograft models of colorectal cancer and to patient studies of prostate cancer and has recently been shown to be feasible in glioma [16–19]. Here, we have applied this method to image intratumoural and intertumoural heterogeneity in glioma and validated with histology as part of a prospective study.

## Methods and materials

### Patient selection

Fourteen consecutive treatment-naïve patients (six men, eight women; age 43.5 years ± 15.1 years) were recruited into this prospective ethically approved study from a neuro-oncology multidisciplinary team meeting or clinic. Subjects were scheduled for stereotactic biopsy or resection and gave written informed consent.

### MRI acquisition

MRI examinations were performed using a 3.0-T clinical scanner and a 12-channel head coil (Discovery MR750; GE Healthcare). Imaging included the following: 3D T2-weighted (T2W) imaging (repetition time/echo time (TR/TE) 2500/79 ms; 248 slices; slice thickness 1.2 mm; acquisition matrix 320 × 320; field of view (FOV) 240 mm × 240 mm), 2D T2W fluid-attenuated inversion recovery (FLAIR) (TR/TE 8000/126 ms; inversion time (TI) 2128 ms; slice thickness 6 mm; acquisition matrix 384 × 224; FOV 240 mm × 240 mm) and DWI (TR/TE 2841–3867/83.3–87.4 ms; slice thickness 5 mm; *b* value 0 and 1000 s/mm^2^; matrix 128 × 128; FOV 240 mm × 240 mm). Three-dimensional T1-weighted fast spoiled gradient echo (T1W FSPGR) (252 slices; 0.94 mm × 0.94 mm × 1.5 mm reconstructed to 0.94 mm × 0.94 mm × 1 mm; TR/TE 8.2/3.2; slice thickness 1.5 mm; matrix 256 × 256; FOV 240 mm × 240 mm) was performed before and after gadolinium-based contrast injection (gadobutrol 1.0 mmol/mL; Schering). ADC maps were generated as per clinical standard.

### VERDICT acquisition and post-processing

The parameters for DWI and VERDICT modelling are shown in Table 1, and the spatial resolution was 2 mm isotropic [17]. For each *b* value, a separate *b* = 0 image was acquired to compensate for varying T2-weighted imaging. To assess the robustness of the five-*b* value–abbreviated acquisition, an extended protocol using 40 *b* values was undertaken in one patient within the limits of the clinical gradient system (*G*_max_, 45 mT/m; slew rate, 200 mT/m/s).

**Table 1:** Diffusion MRI parameters for the abbreviated VERDICT model

*b* value (s/mm^2^)	∆/*∂* (ms)	Echo time (ms)	|*G*| (mT/m)	Number of averages	Acceleration factor	Full readout bandwidth (Hz)
90	23.5/4.7	49.3	49.4	8	2	200,000
500	31.3/12.2	64.3	41.5	8	2	200,000
1500	43.4/25.8	91.4	30.1	8	2	200,000
2000	32.1/16.5	72.7	75.8	8	2	200,000
3000	43.8/24.8	89.4	43.9	8	2	200,000

The following are the parameters used for the shortened protocol: ∆ is the time separation between the gradient pulses, *∂* is the duration of the pulses and |*G*| is the combined gradient strength of the diffusion encoding

The tumours were located on T2W, and then 16 axial DWI slices were acquired over the region of interest (ROI) for VERDICT modelling. The acquisition time for each *b* value was 66 s, with a total time of 330 s. Tumour cell radius, intracellular (IC) and combined extracellular (EC)/vascular volume were calculated (Matlab 2016b, the MathWorks) using a framework based on linearisation and convex optimisation which provides an acceleration factor of 1500 [20, 21]. Intracellular and extracellular spaces were modelled separately, and therefore, the sum of both compartments was only approximately 100%. Parameter maps were registered to post-contrast 3D T1W and 3D T2W sequences (SPM12, UCL).

### MRI analysis

A neuroradiologist with 6 years of experience in neuro-imaging outlined ROIs on axial T2W and axial 3D T1W post-contrast sequences using OsiriX (V.8.5.2, Pixmeo SARL). To assess the reproducibility of the ROI definition, a second observer with 3 years of experience in neuro-imaging, blind to the clinical data, performed a second analysis using the same software. ROIs were drawn around the entire lesion including the surrounding oedema using a combined evaluation of T1W and T2W imaging. The 3D T1W post-contrast images were used to better define the enhancing tissue and the necrosis (if present) whilst the T2W images were used to better highlight the oedema boundaries. ADC and VERDICT maps were co-registered to the anatomical imaging to calculate the microstructural parameters.

### Pathological analysis

Histopathology was used to determine the tumour grade and type using the WHO 2016 classification [1]. A neuropathologist with 7 years of experience evaluated tumour photomicrographs from a × 600 FOV and a fixed matrix of 184 μm × 138 μm to assess cellularity and cell size using semi-automated software (Image-Pro Insight). Average tumour cell size was assessed by measuring the short and long axes of 20 cancer cells in each FOV. To determine if shrinkage post-fixation affected these measurements, red blood cell size was compared between a blood smear and a stained section in the same patient [22, 23].

### Statistical analysis

Statistical analysis was performed using a mathematical analytical software program (Matlab Statistics and Machine Learning Toolbox, Matlab 2017a, MathWorks). The Kolmogorov–Smirnov test was used to test for normality of data. Comparisons between pathological analysis and VERDICT parameters were performed using the Wilcoxon rank-sum test. The same non-parametric statistical test was used to assess the difference in cell size and density and to compare the results obtained by the two observers. A statistical significance of a *p* value ≤ 0.05 was used. The Dice similarity coefficient (DSC) was used to assess the reproducibility of the segmentation performed by the two observers [24]; a coefficient > 0.70 indicates a good overlap between the ROIs [25, 26].

## Results

The demographics and tumour characteristics are shown in Table 2. VERDICT fitting was successfully performed for all patients. The time window between imaging and surgery was 17.5 ± 12.3 (median ± SD; range 4–46) days in patients with low-grade glioma (LGG) and 5.2 ± 6.4 (1–17) days in patients with high-grade glioma (HGG). The following patients were included: seven with LGG (four diffuse, isocitrate dehydrogenase 1 (IDH1) R132H mutant astrocytomas; three IDH1 R132H mutant, 1p19q co-deleted oligodendrogliomas) and seven with HGG (six IDH wild-type glioblastomas; one IDH wild-type astrocytoma).

**Table 2:** Demographic data and histopathological characteristics for high-grade and low-grade tumours

	Low-grade glioma	High-grade glioma	*p*
Age in years	33.2 ± 9.2 (24–45)	55.3 ± 11.9 (44–78)	< 0.01
Men, *n* (%)	2 (28.6)	4 (57.1)	0.59
Lesion size in mm	44.4 ± 19.1 (23.1–68.7)	27.1 ± 17.2 (6.4–56.4)	0.13
IDH1 R132H^a^, *n* (%)			
Present	7 (100)	0 (0)	< 0.01
Absent	0 (0)	7 (100)	
MIB-1 (a.u.)	4.2 ± 3.0 (1–8.8)	19.2 ± 6.7 (12–31)	< 0.001

The monoclonal antibody MIB-1 was used to determine the Ki-67 labelling index as a marker of proliferation. Continuous values are expressed as mean ± SD with range in parentheses

^a^Mutations involving isocitrate dehydrogenase 1

### Histopathological analysis

**Fig. 1:**
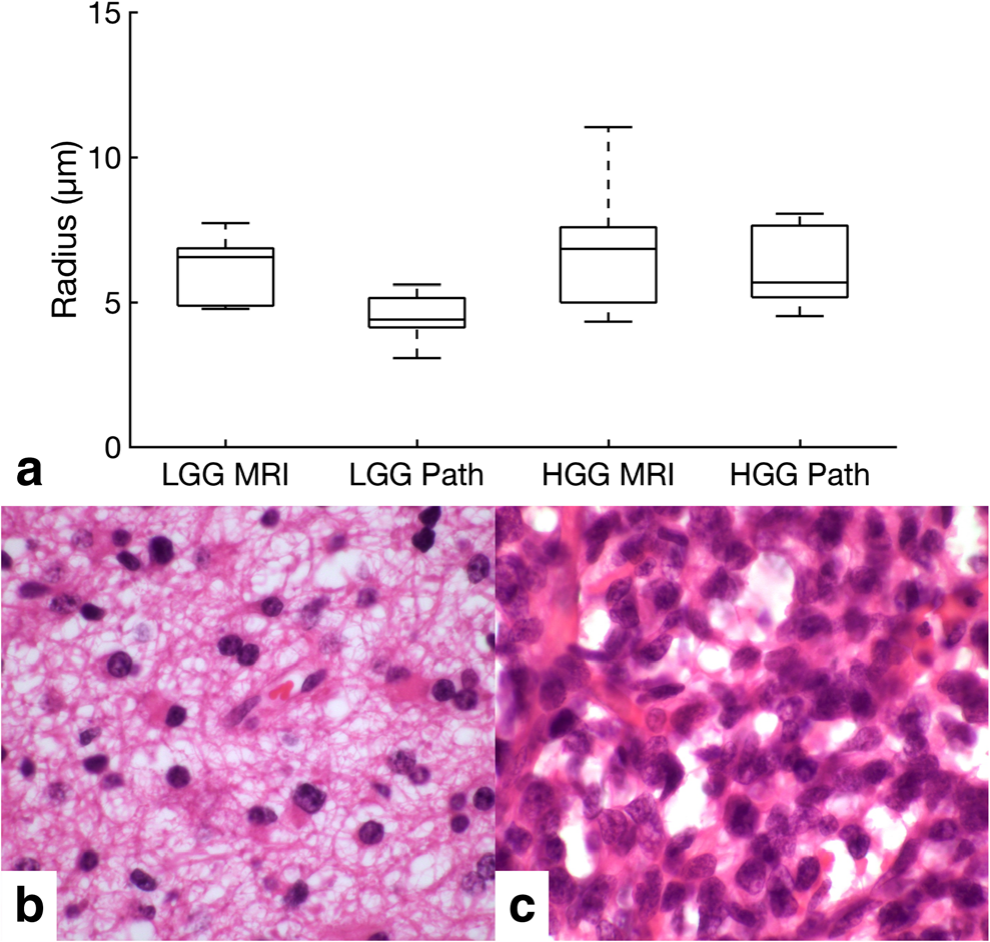
**a** Box and whisker plot illustrating the cell radius as measured by VERDICT MRI and pathology for low-grade glioma (LGG) and high-grade glioma (HGG). **b** H&E staining from a low-grade glioma shown in Fig. 2. **c** H&E staining from a high-grade glioma also shown in Fig. 2

The histopathological measure of cell radius (Fig. 1) was not significantly different in HGG compared to LGG (5.7 ± 1.4 μm (range 4.5–8.1) vs. 4.6 ± 1.6 μm (3.1–8.1), respectively (*p* = 0.16)). The red blood cell size was 5.5 ± 0.7 μm (range 4.4–6.9) on fixed sections and 7.6 ± 0.6 μm (6.6–8) on the cell smear (*p* < 0.001), demonstrating a reduction in size following fixation.

There was a trend towards a higher histological cell density in HGG compared to LGG (104 ± 43 cells/FOV (range 67–170) vs. 51 ± 66.4 cells/FOV (35–210), respectively); the difference was not significant (*p* = 0.21).

### VERDICT analysis

**Fig. 2:**
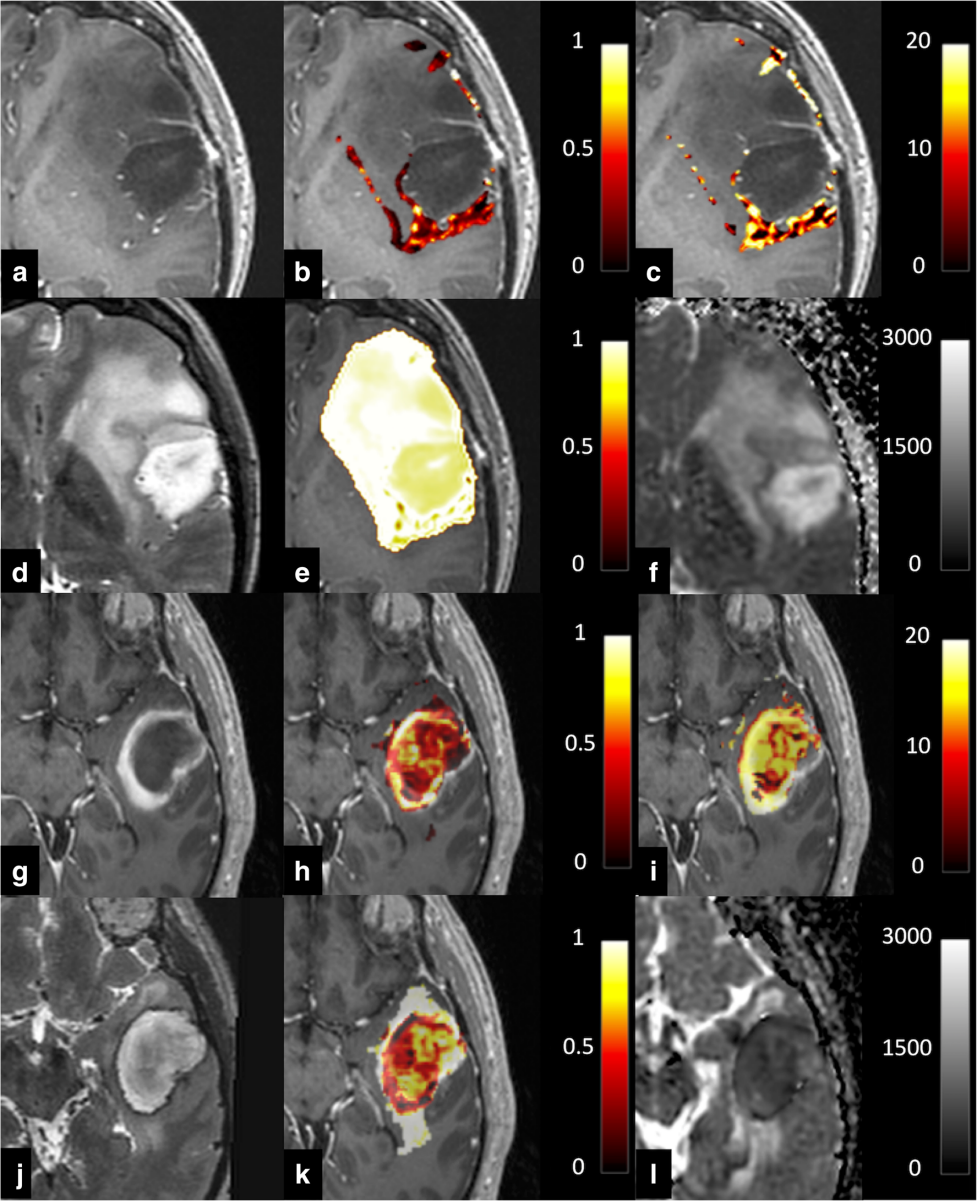
**a**–**f** Representative images from a low-grade glioma. **a** Axial post-gadolinium 3D T1-weighted imaging (T1WI). **b** Intracellular volume fraction. **c** Cell radius maps. **d** Axial T2WI. **e** Extracellular volume fraction. **f** ADC map, with a scale of × 10^−6^ mm^2^/s. Colour maps for **b**, **c**, **e** have been superimposed on the greyscale image from **a** with the colour scale shown for each image. **g**–**l** Representative images from a high-grade glioma. **g** Axial post-gadolinium 3D T1WI. **h** Intracellular volume fraction. **i** Cell radius maps. **j** Axial T2WI. **k** Extracellular volume fraction. **l** ADC map, with a scale of × 10^−6^ mm^2^/s. Colour maps for **h**, **i**, **k** have been superimposed on the greyscale image from **a** with the colour scale shown for each image

**Fig. 3:**
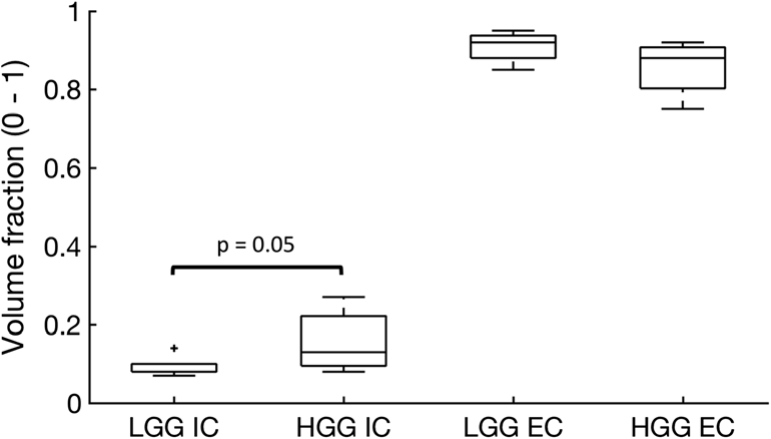
Box and whisker plot showing intracellular (IC) volume fraction and extracellular (EC) volume fraction for both low-grade glioma (LGG) and high-grade glioma (HGG) derived from the VERDICT MRI model

**Fig. 4:**
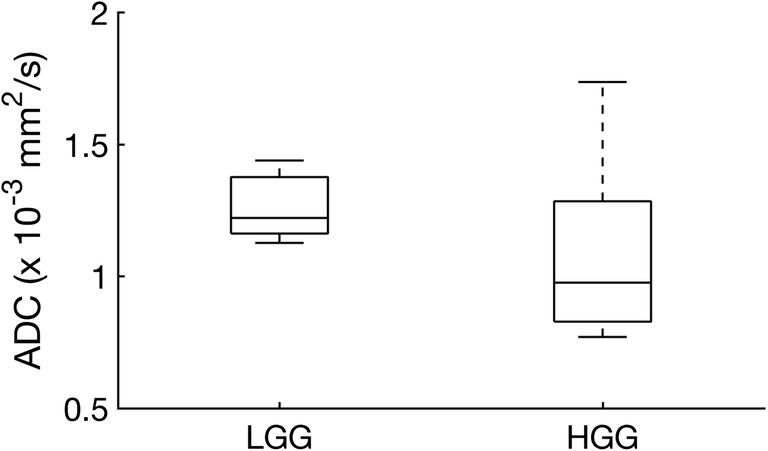
Apparent diffusion coefficient (ADC) values for low-grade glioma (LGG) and high-grade glioma (HGG) as derived from conventional DWI

VERDICT parametric maps for LGG and HGG are shown in Fig. 2. The calculated cell radius was similar in both groups (6.8 ± 2.3 μm for HGG (range 4.3–11) and 6.7 ± 1.2 μm for LGG (4.8–7.8); Fig. 1). There were no significant differences between VERDICT radius measurements and those from pathological sections for either LGG (*p* = 0.09) or HGG (*p* = 0.80). The intracellular volume fraction was higher in HGG compared to LGG (0.13 ± 0.07 (range 0.08–0.27) vs. 0.08 ± 0.02 (0.07–0.14), respectively; *p* = 0.05). The extracellular/vascular volume fraction showed the opposite trend with a higher EC volume fraction in LGG compared to HGG, but this was not significant (0.92 ± 0.04 (range 0.85–0.95) vs. 0.88 ± 0.07 (0.75–0.92), respectively; *p* = 0.10; Fig. 3). DWI as per clinical standard was obtained in 12/15 patients. The conventional ADC was higher in LGG compared to HGG (1.22 ± 0.13 × 10^−3^ mm^2^/s (range 1.13–1.44) vs. 0.98 ± 0.38 × 10^−3^ mm^2^/s (0.77–1.74)); however, the difference was not statistically significant (*p* = 0.18; Fig. 4).

### Reproducibility analysis

The assessment of the reproducibility in the ROI segmentation for the entire tumour region performed by the two observers resulted in high reproducibility demonstrated by a DSC of 0.89 ± 0.06 (0.80–0.99). A sub-analysis performed according to grading demonstrated similar results with a DSC of 0.89 ± 0.05 (0.82–0.94) for LGG and 0.89 ± 0.08 (0.80–0.99) for HGG.

The results of the analysis performed by the second observer are shown in Table 3.

**Table 3:** Comparison between the two observers

	LGG			HGG		
	Obs 1	Obs 2	*p*	Obs 1	Obs 2	*p*
Cell radius (μm)	6.7 ± 1.2	6.7 ± 1.5	0.53	6.8 ± 2.3	6.1 ± 2.1	1
IC volume fraction	0.08 ± 0.02	0.11 ± 0.03	0.07	0.13 ± 0.07	0.12 ± 0.06	0.93
EC volume fraction	0.92 ± 0.04	0.91 ± 0.05	0.60	0.88 ± 0.07	0.89 ± 0.05	0.94

*Obs* observer

The comparison between the VERDICT parameters derived by the analysis performed by the two observers. Values are expressed as median ± SD

## Discussion

VERDICT is an advanced diffusion method which has previously been applied to pre-clinical models of cancer showing microstructural differences between tumours and a decrease in cell volume following chemotherapy [16]. The method has also been shown to differentiate benign prostate tissue from tumour in patients [17]. In this study, we have compared VERDICT to histopathological findings at surgery and correlated with tumour grade. Non-invasive grading of glioma is a major challenge for conventional MRI, and biopsied tissue is limited by sampling error as the tumour is very heterogeneous [9]. Conventional ADC has a sensitivity and specificity for differentiating high- and low-grade tumours of only 85% and 80%, respectively, and there is a large overlap in ADC between grades [27]. Therefore, additional imaging methods are required to accurately differentiate tumour subtypes and to demonstrate tumour heterogeneity [12].

VERDICT parameters showed a higher intracellular volume fraction in HGG compared to LGG and a trend towards a smaller extracellular/vascular volume fraction. As IC and EC volume fractions are independently derived, their complementarity demonstrates intramodel consistency. Moreover, these results were validated with the histological findings where there was a higher cell density in HGGs compared to LGGs. Importantly, although the conventional ADC was higher in LGG compared to HGG, this was not statistically significant. Therefore, VERDICT could provide important additional information which is complementary to conventional DWI.

VERDICT-derived maps showed a higher degree of heterogeneity compared to the corresponding ADC maps. This phenomenon was particularly evident in HGG (Fig. 4) where regional differences in the VERDICT maps were not visible on ADC. The areas of high IC volume largely corresponded to the independently derived areas of low EC volume, which again provides evidence that the model is internally consistent. Future studies can validate this heterogeneity using multiregional biopsies, and the technique could be used as a tool to assess changes in this heterogeneity with treatment as well as a method to assess the resection cavity following surgery [16].

The range of cell size was larger for HGG compared to LGG on both VERDICT and histopathology in keeping with the more heterogeneous nature of the higher-grade tumours. The slight reduction in cell size measurements on histopathology compared to the in vivo measurements may be accounted for by shrinkage following fixation [22, 23]. Despite the correlation between overall cell size on imaging and histology, the patient-by-patient validation against pathology demonstrated no statistically significant difference which may be accounted for by sampling error, given that only a small proportion of the tumour was biopsied and this may not represent the whole tumour [16].

Rapid acquisition and data analysis are necessary for clinical translation of the method, and the abbreviated VERDICT MRI protocol proposed by Panagiotaki et al [28] was performed here in 5.5 min, representing a reduction of 45 min compared to the extended protocol. In addition, the data fitting was combined with a post-processing algorithm based on linearisation and convex optimisation which reduced the computing by more than 1500-fold to allow the analysis to be made available within a clinically applicable timescale (25.5 min ± 5.3 min) for acquisition and post-processing [20, 21].

The primary objective of the work was to assess the feasibility of using VERDICT MRI to investigate the tissue microstructure in glioma in a clinical setting. The limitations of the study include a small sample size which may have reduced the statistical power of the study. Also, the original VERDICT model is designed to compute the intracellular, intravascular and extracellular–extravascular spaces in an animal model of prostate cancer at a pre-clinical field strength of 9.4 T which has significantly higher diffusion gradient capabilities of ~ 300 mT/m. The computational modelling can become unstable with the noise and time constraints experienced at clinical field strength which can compromise the computational fit of the intravascular compartment [17]. The correlation between histology and imaging is also challenging as 2-mm-thick imaging slices were compared to 4-μm pathological slides which could introduce sampling errors, particularly given the heterogeneous nature of glioma. Future larger studies are needed to more fully validate the technique against histopathology and to explore its potential applications as a routine clinical tool.

In conclusion, VERDICT MRI could be a promising technique to assess the tissue microenvironment within glioma using a clinically applicable protocol. The derived parameters demonstrated larger cells and a trend towards a smaller extracellular space in high-grade tumours compared to low-grade tumours. This approach may provide additional information compared to conventional diffusion-weighted imaging.

## Electronic supplementary material

ESM 1 (DOCX 17 kb)

## Abbreviations

ADC: Apparent diffusion coefficient
DWI: Diffusion-weighted imaging
HGG: High-grade glioma
IDH1: Isocitrate dehydrogenase 1
LGG: Low-grade glioma
MRI: Magnetic resonance imaging
T1W: T1-weighted
T2W: T2-weighted
VERDICT: Vascular, extracellular and restricted diffusion for cytometry in tumours

## References

[1] Louis DN, Perry A, Reifenberger G (2016). The 2016 World Health Organization classification of tumors of the central nervous system: a summary. Acta Neuropathol.

[2] Paulus W, Peiffer J (1989). Intratumoral histologic heterogeneity of gliomas. A quantitative study. Cancer.

[3] Daher A, de Groot J (2018). Rapid identification and validation of novel targeted approaches for glioblastoma: a combined ex vivo-in vivo pharmaco-omic model. Exp Neurol.

[4] Anjum K, Shagufta BI, Abbas SQ (2017). Current status and future therapeutic perspectives of glioblastoma multiforme (GBM) therapy: a review. Biomed Pharmacother.

[5] Mabray MC, Barajas RF Jr, Cha S (2015) Modern brain tumor imaging. Brain Tumor Res Treat 3:8–23 10.14791/btrt.2015.3.1.8 PMC4426283 25977902

[6] Ceccarelli M, Barthel FP, Malta TM (2016). Molecular profiling reveals biologically discrete subsets and pathways of progression in diffuse glioma. Cell.

[7] Aihara K, Mukasa A, Nagae G (2017). Genetic and epigenetic stability of oligodendrogliomas at recurrence. Acta Neuropathol Commun.

[8] Sottoriva A, Spiteri I, Piccirillo SG (2013). Intratumor heterogeneity in human glioblastoma reflects cancer evolutionary dynamics. Proc Natl Acad Sci U S A.

[9] Langen KJ, Galldiks N, Hattingen E, Shah NJ (2017). Advances in neuro-oncology imaging. Nat Rev Neurol.

[10] Bulakbasi N, Guvenc I, Onguru O, Erdogan E, Tayfun C, Ucoz T (2004) The added value of the apparent diffusion coefficient calculation to magnetic resonance imaging in the differentiation and grading of malignant brain tumors. J Comput Assist Tomogr 28:735–746 10.1097/00004728-200411000-00003 15538145

[11] Hilario a, Sepulveda JM, Perez-Nuñez a (2014). A prognostic model based on preoperative MRI predicts overall survival in patients with diffuse gliomas. AJNR Am J Neuroradiol.

[12] Kono K, Inoue Y, Nakayama K (2001). The role of diffusion-weighted imaging in patients with brain tumors. AJNR Am J Neuroradiol.

[13] Lee EJ, terBrugge K, Mikulis D (2011). Diagnostic value of peritumoral minimum apparent diffusion coefficient for differentiation of glioblastoma multiforme from solitary metastatic lesions. AJR Am J Roentgenol.

[14] Oh J, Cha S, Aiken AH (2005). Quantitative apparent diffusion coefficients and T2 relaxation times in characterizing contrast enhancing brain tumors and regions of peritumoral edema. J Magn Reson Imaging.

[15] Server A, Kulle B, Mæhlen J (2009). Quantitative apparent diffusion coefficients in the characterization of brain tumors and associated peritumoral edema. Acta Radiol.

[16] Panagiotaki E, Walker-Samuel S, Siow B (2014). Noninvasive quantification of solid tumor microstructure using VERDICT MRI. Cancer Res.

[17] Panagiotaki E, Chan RW, Dikaios N (2015). Microstructural characterization of normal and malignant human prostate tissue with vascular, extracellular, and restricted diffusion for cytometry in tumours magnetic resonance imaging. Invest Radiol.

[18] Bailey C, Collins DJ, Tunariu N (2018). Microstructure characterization of bone metastases from prostate cancer with diffusion MRI: preliminary findings. Front Oncol.

[19] Roberts T, Hyare H, Hipwell B (2018). Quantification of tumour microstructure in low and high-grade brain tumours using VERDICT MRI: an initial feasibility study. Neuro-Oncol.

[20] Daducci A, Canales-Rodríguez EJ, Zhang H (2015). Accelerated microstructure imaging via convex optimization (AMICO) from diffusion MRI data. Neuroimage.

[21] Bonet-Carne E, Daducci A, Panagiotaki E et al (2016) Non-invasive quantification of prostate cancer using AMICO framework for VERDICT MR. In: International Society for Magnetic Resonance in Medicine (ISMRM). pp 5–8

[22] Tran T, Sundaram CP, Bahler CD (2015). Correcting the shrinkage effects of formalin fixation and tissue processing for renal tumors: toward standardization of pathological reporting of tumor size. J Cancer.

[23] Hsu PK, Huang HC, Hsieh CC (2007). Effect of formalin fixation on tumor size determination in stage I non-small cell lung cancer. Ann Thorac Surg.

[24] Dice LR (1945). Measures of the amount of ecologic association between species. Ecology.

[25] Zijdenbos AP, Dawant BM, Margolin RA, Palmer AC (1994) Morphometric analysis of white matter lesions in MR images: method and validation. IEEE Trans Med Imaging 13:716–724 10.1109/42.363096 18218550

[26] Zou KH, Warfield SK, Bharatha A (2004). Statistical validation of image segmentation quality based on a spatial overlap index. Acad Radiol.

[27] Zhang L, Min Z, Tang M, Chen S, Lei X, Zhang X (2017) The utility of diffusion MRI with quantitative ADC measurements for differentiating high-grade from low-grade cerebral gliomas: evidence from a meta-analysis. J Neurol Sci 375:103–106 10.1016/j.jns.2016.12.008 28131237

[28] Panagiotaki E, Ianus A, Johnston E et al (2015) Optimised VERDICT MRI protocol for prostate cancer characterisation. In: Proceedings of the 23rd meeting of the International Society for Magnetic Resonance in Medicine 2015. p 2872

