# Supplementary material for: Non-invasive assessment of glioma microstructure using VERDICT MRI: correlation with histology
Source: Eur Radiol. 2019 Mar 19;29(10):5559–66. doi: 10.1007/s00330-019-6011-8 (PMC6719328; doi:10.1007/s00330-019-6011-8)
Supplement: Supplementary file 1 — (DOCX 17 kb) [file 330_2019_6011_MOESM1_ESM.docx]

**Supplementary material**

*Short vs. extended protocol*

The extended protocol duration was 49.5 min and the post-processing required 16 min; the abbreviated protocol was 5.5 min and the post-processing required 20±5.3 min (Table 4). The computing time to perform the fit without linearization and convex optimization was estimated to be more than 343 hours for a single patient and was not deemed feasible. The cell radius was 4.1 ± 0.6 µm using the extended protocol and 3.4 ± 0.7 µm with the abbreviated protocol; the average cell radius at pathology was 4.1 ± 1.3 µm.

|  | **Extended protocol** | **Abbreviated protocol** |
| --- | --- | --- |
| **Scan time (min)** | 49.5 | 5.5 |
| **Cell radius (µm)** | 4.1 ± 0.6 | 3.4 ± 0.7 |
| **IC volume fraction** | 0.12 ± 0.02 | 0.11 ± 0.01 |
| **EC volume fraction** | 0.88 ± 0.02 | 0.81 ± 0.02 |

**Table 4:** Extended VERDICT MRI protocol compared to the abbreviated protocol.

Comparison between the scanning time and derived metrics for the full and the abbreviated protocols for the single patient that underwent both approaches. Values are expressed as mean ± SD.
